# The Detection of Malingering: A New Tool to Identify Made-Up Depression

**DOI:** 10.3389/fpsyt.2018.00249

**Published:** 2018-06-08

**Authors:** Merylin Monaro, Andrea Toncini, Stefano Ferracuti, Gianmarco Tessari, Maria G. Vaccaro, Pasquale De Fazio, Giorgio Pigato, Tiziano Meneghel, Cristina Scarpazza, Giuseppe Sartori

**Affiliations:** ^1^Department of General Psychology, University of Padova, Padova, Italy; ^2^Department of Human Neurosciences, University of Roma “La Sapienza”, Rome, Italy; ^3^Neuroscience Center, Department of Medical and Surgical Science, University “Magna Graecia”, Catanzaro, Italy; ^4^Department of Psychiatry, University “Magna Graecia”, Catanzaro, Italy; ^5^Psychiatry Unit, Azienda Ospedaliera di Padova, Padova Hospital, Padova, Italy; ^6^Dipartimento di Salute Mentale, Azienda Unità Locale Socio Sanitaria 9, Treviso, Italy

**Keywords:** depression, malingering, decetpion, machine learning, automatic

## Abstract

Major depression is a high-prevalence mental disease with major socio-economic impact, for both the direct and the indirect costs. Major depression symptoms can be faked or exaggerated in order to obtain economic compensation from insurance companies. Critically, depression is potentially easily malingered, as the symptoms that characterize this psychiatric disorder are not difficult to emulate. Although some tools to assess malingering of psychiatric conditions are already available, they are principally based on self-reporting and are thus easily faked. In this paper, we propose a new method to automatically detect the simulation of depression, which is based on the analysis of mouse movements while the patient is engaged in a double-choice computerized task, responding to simple and complex questions about depressive symptoms. This tool clearly has a key advantage over the other tools: the kinematic movement is not consciously controllable by the subjects, and thus it is almost impossible to deceive. Two groups of subjects were recruited for the study. The first one, which was used to train different machine-learning algorithms, comprises 60 subjects (20 depressed patients and 40 healthy volunteers); the second one, which was used to test the machine-learning models, comprises 27 subjects (9 depressed patients and 18 healthy volunteers). In both groups, the healthy volunteers were randomly assigned to the liars and truth-tellers group. Machine-learning models were trained on mouse dynamics features, which were collected during the subject response, and on the number of symptoms reported by participants. Statistical results demonstrated that individuals that malingered depression reported a higher number of depressive and non-depressive symptoms than depressed participants, whereas individuals suffering from depression took more time to perform the mouse-based tasks compared to both truth-tellers and liars. Machine-learning models reached a classification accuracy up to 96% in distinguishing liars from depressed patients and truth-tellers. Despite this, the data are not conclusive, as the accuracy of the algorithm has not been compared with the accuracy of the clinicians; this study presents a possible useful method that is worth further investigation.

## Introduction

Major depression is a high-prevalence [7%; (1)] mental disease with major socio-economic impact for both direct (medications and hospitalization) and indirect (mortality, work absence and turnover, disability compensation) costs (2). Strikingly, the length of absence from work due to depressive disorder is significantly longer than that due to organic serious illnesses such as heart disease, back pain, diabetes mellitus and hypertension (3). Although important, absenteeism is not the only cost whereby depression burdens the public health, as a critical percentage of the national health system income is devolved for the provision of invalidity pensions. The Italian government, for instance, recognizes an invalidity of up to 80% for people suffering with endogenous depression, with the consequent allocation of monthly disability checks amounting from 270 to 500 euro per person (4). In addition, in Italy, insurance companies spend weighty annual sums for the compensation of psychic damage, including depression, which could result, for example, from road accidents, stalking and mobbing (5).

Due to the undeniable economic advantages of being clinically depressed, major depression symptoms can be faked or exaggerated in order to obtain economic compensation. The literature on this topic is still at its infancy (6). In Italy, the problem of people feigning a wide range of symptoms to obtain disability pensions is of critical relevance. Indeed, in some regions of Italy, people feigned many conditions, from inability to walk to blindness to obtain economic advantages (https://www.ilfattoquotidiano.it/2013/02/09/falsi-invalidi-meccanico-in-svizzera-da-2500-prendeva-pensione-da-1300-in-italia/494390/; http://www.iltempo.it/economia/2016/03/31/news/tre-milioni-di-invalidi-100-mila-falsi-1005788/). Critically, the malingered conditions are simple to be feigned. The same might be true for depression; its symptoms are very intuitive for naïve people (7), as everyone has experienced low mood during life. Importantly, experienced clinicians are trained not to only rely on the self-reported symptoms provided by patients. Indeed, according with the DSM-5 guidelines, outstandingly important pieces of information also came from direct observation of signs of depression, observation of the non-verbal behavior of the patients, and convergence of the information reported by patients and relatives. However, the behavioral observations rely heavily on information that could be consciously controlled by the patient. This is because, as depression is a very common disorder, individuals who want to feign a depressive disorder do not require any particular knowledge or specific training to produce clinically reliable depressive symptoms and signs. Furthermore, a large majority of both symptoms and signs easy to fake: lack of concentration, restlessness, lack of interest for daily life activities, feelings of guilt, and so on are easy to fake if one wanted and planned to. For this reason, depression is one of the mental disorders that are more frequently and easily faked to achieve financial or other advantages, and this underlines, in the forensic setting, the necessity to couple the psychiatric examination with a different methodology, which is less influenced by the individuals' overt decisions to malinger a psychiatric disorder.

Malingering is defined as the voluntary fabrication or exaggeration of mental or physical symptoms to gain secondary benefits, which could include financial compensations or other advantages, such as leniency, drugs, avoiding obligations (school, work, army), or just getting the attention of other people (8).

Although malingering is not considered to be a mental disorder, recent scientific knowledge suggest that it should be the focus of clinical attention, so much so that it has been introduced in the Diagnostic and Statistical Manual of Mental Disorders [DSM-5; (1)]. This has been an important step forward in the scientific community, as the fabrication/accentuation of symptoms or the concealment of a disorder are very frequent, especially when the evaluation takes place in forensic contexts (9). Although it is hard to define it reliably, literature reports an estimate of the prevalence of malingering in a forensic setting as ranging from 20 to 40%. (9–11). In regards to depression, Mittenberg et al. reported that 16.08% of depressive syndromes which are diagnosed in litigation or compensation cases are feigned (10).

Currently, a diagnosis of a psychiatric disorder, including depression, is formulated according to the subjective experience that is reported by the patient and to the observation of signs and non-verbal behavior that are easily manipulated by the individual will to deceive (12). In other words, the psychic symptom exists because the patient refers to it, and the assessment of malingering is mostly based on clinical judgment (13). While this aspect is less (or none) than a problem in the clinical setting, in which patients are seeking help for their sufferance and in which malingering itself become a symptom (as in the case of factious disorder or Munchausen syndrome), the forensic context is a quite different situation. Indeed, as already introduced, in the psychiatric setting, symptoms can be exaggerated or faked to obtain a secondary advantage. Thus, classical psychiatric or clinical evaluation itself is not reliable when dealing with forensic-relevant topic. The limitations of classic psychiatric evaluation alone have been provocatively investigated in a well-known experiment conducted by Rosenhan (14), in which “pseudo-patients” feigning hallucinations were all admitted to the psychiatric department of 12 different highly specialized hospitals: all but one (who was diagnosed as having a bipolar disorder) received a clinical diagnosis of full-blown schizophrenia. In another study (15), the authors reported that experienced psychiatrists distinguished actors and depressed patients during a clinical interview with an accuracy close to the chance level. Furthermore, the clinicians rated their confidence in their diagnoses as 6.5 out of 10 in the case of patients and 7.1 in the case of actors, denoting that they were equally certain of their right and wrong diagnoses. Considered together, the results of these studies highlighted the urgent need to have complementary and integrative tools that may strengthen the process of achieving a correct psychiatric diagnosis.

This low reliability of the classical clinical evaluation used alone in detecting malingering in forensic setting led to an exponential growth of the research in this topic over the last 15 years (16). Different strategies to detect malingering in clinical and forensic setting have been proposed, and *ad hoc* tests have been developed. Strategies are varied, but until recently, they were mainly based on self-report questionnaires such as the M-Test and the Structured Inventory of Malingered Symptomatology (SIMS). The former was conceived to be specifically applied to the feigning of schizophrenic symptoms (17), while the latter was conceived to detect malingering of both psychiatric syndromes, including depression, and cognitive deficits (18). Although these instruments have been undoubtedly useful, they share the important limitation of the psychiatric assessment as described above: they are based on the “patients”' report of symptoms, and they can be easily deceived by coaching (19). As psychiatric symptoms are easy to be feigned, unmasking the simulation of psychiatric syndromes is much more challenging than unmasking other pathologies (e.g., cognitive disorders), and thus the instrument to detect them should be more sophisticated.

An important advance over the self-report questionnaires has been achieved in the last few years, as behavioral-based lie detection techniques to spot the simulation of psychiatric symptoms have been introduced. Contrary to the self-report questionnaire, which took into consideration the explicit answers of subjects, the behavioral methods mainly rely on implicit measures not fully under the explicit and conscious control of the evaluated subject. For example, the autobiographical Implicit Association Test [aIAT; (20)] and the Concealed Information Test [CIT; (21)] are able to identify liars based on their response time (TR). Concerning malingering detection, the aIAT has been successfully applied to detect whiplash malingering, confirming an accuracy of around 90% (22), as well as to unveil phantom-limb pain (13) and psychogenic amnesia (16). The CIT has been principally used to assess the simulation of amnesia (21). The main limitation of these implicit tools is that they can only investigate one symptom at a time. In other words, more than one aIAT or CIT would be necessary to establish whether the subject is feigning a psychiatric syndrome or not, checking all of the symptoms, one by one, with a specific test.

Interestingly, different studies in literature have shown that deception can be captured through analysis of hand-motor responses while the subject is engaged in a double-choice task (23–26). More in general, the kinematic analysis can be used as an implicit online measure of the mental operations that are put in place by the subject during a task (27). A simple hand movement, such as the movement of the mouse on the computer screen, reflects, in real time, the evolution of the cognitive processes underlying the action. Because lying requires great cognitive resources (28), the motor response to a stimulus is altered in terms of spatial and temporal features compared to the truth telling (23, 26).

The aim of the present study is to present a new tool specifically developed to detect malingering, which has the important advantages of: (i) being conceived specifically to evaluate the truthfulness of depressive syndrome (but that might also be adapted to other psychopathologies); (ii) relying on an implicit measure, i.e. the kinematic of movement, which is not consciously controllable; (iii) being not easy to cheat by knowing the symptomatology or by coaching; (iv) being based on machine learning algorithm that will allow the identification of liars at individual level; and (v) considering at the same time both implicit variables and clinical symptoms.

## Methods

### Participants

As machine-learning algorithms require to be built and tested using two independent samples, two independent groups of participants have been selected for this research.

#### Group 1

Seventy-two Italian-speaking participants were recruited, with the aim to build machine-learning classification models. In detail, 26 patients suffering from depression were recruited (see below for details), as well as 46 age- and gender-matched healthy volunteers.

Before the experiment, the Beck Depression Inventory [BDI, (29)] was administered to all participants with the aim to exclude possible sub-clinical participants (undiagnosed depressed participants) within the healthy controls and to exclude responder participants (defined as clinically diagnosed participants under medications who did not manifest depressive symptoms) from the clinical group. Six sub-clinical participants and six responders were identified and excluded from the experiment. The final sample consisted of 60 participants (39 females, 21 males). The average age was 38.60 years (*SD* = 14.74), and the average education level was 15.15 years (*SD* = 2.98).

Twenty participants were depressed patients, and the remaining 40 individuals were healthy subjects randomly assigned to the truth-teller (e.g., non-depressed participants who were instructed to respond truthfully to the test; *n* = 20) or liar (e.g., non-depressed participants who were instructed to respond deceitfully to the test; *n* = 20) condition. An ANOVA confirmed that the three groups were similar in terms of age and schooling (*p* > 0.01 for both age and schooling), whereas a Chi-squared test (χ^2^) confirms that they were similar also for gender (all *ps* > 0.01). On the contrary, the groups differed in the BDI score [*F*_(2, 57)_ = 83.41, *p* < 0.01]: the post hoc test highlighted that, tautologically, the truth-tellers' BDI average score of 6.1 (*SD* = 3.97) was similar to the average liars' score of 6.2 (*SD* = 3.62), while the BDI score of the depressed patients clearly differed from the one of the healthy volunteers score of 29.5 (*SD* = 10.09).

The patients suffering from depression were recruited from Azienda Ospedaliera Sant'Andrea di Roma (*n* = 4), Ospedale Ca' Foncello di Treviso (*n* = 10), Unità Operativa di Psichiatria, Dipartimento di Scienze della Salute dell'Università Magna Grecia (Catanzaro; *n* = 5), and Azienda Ospedaliera di Padova (*n* = 1). These patients were diagnosed according to the DSM-IV criteria by an expert psychiatrist at each site. At the time of the study, all of the depressed participants were under pharmacological medications, and seven of them were attending psychotherapy.

#### Group 2

A second group comprising 27 Italian-speaking participants was also enrolled with the aim to test the model and its generalization (30). This second group consisted of 8 males and 19 females with an average age of 35.37 years (*SD* = 21.42) and an average education level of 13.15 years (*SD* = 3.59) and did not statistically differ from Group 1 in any demographic data (age: *p* > 0.01, education: *p* > 0.01). No sub-clinical or responder participants have been identified in this second group. As for Group 1, the participants enrolled in group 2 comprised 9 depressed patients (which were recruited from the same Institutions and with the same modalities of Group 1) and 18 healthy participants. Again, the healthy participants were randomly assigned to the truth-teller (*n* = 9) or liar (*n* = 9) condition. The three groups did not differ in age (*p* > 0.01), schooling (*p* > 0.01), or gender (all *ps* > 0.01), while they differ in BDI score [*F*_(2, 24)_ = 34.65, *p* < 0.01], with the depressed patients scoring higher than the healthy participants (truth-tellers: *M* = 5.8, *SD* = 4.02; liars: *M* = 5.8, *SD* = 3.67; depressed: *M* = 27.9, *SD* = 9.87). All of the depressed participants were under pharmacological medications, and two were in psychotherapy treatment.

All of the participants provided informed consent before the experiment. The experimental procedures were approved by the ethics committee for psychological research of the University of Padova and were in accordance with the declaration of Helsinki and its later amendments.

### Stimuli

The stimuli adopted in the current study consisted of simple and complex questions about symptoms of depression and concerning the experimental condition. Please see Monaro et al. (31) for a description. The typical symptoms of depression were extracted from the Depression Questionnaire (QD) of the Cognitive Behavioural Assessment 2.0 [CBA 2.0; (32)] and from the Structured Clinical Interview for Mood Spectrum [SCI MOODS; (33)].

Simple questions referred to only one piece of information related to the experimental condition (e.g., “Are you carrying out a questionnaire?”) or one piece of information related to a single symptom of depression (e.g., “Do you feel tired very easily?”). Each simple question required a “yes” or “no” response. Contrarily, complex questions are questions which comprised two (or more) pieces of information. A complex question required a “yes” response when both pieces of information were true, whereas it requires a “no” response when at least one of the two pieces of information was false. Asking complex questions is a method used to overcharge the cognitive load of liars (34). In fact, literature showed that the increment of the liar's cognitive load is an effective strategy to spot deceptive responses (35). While a truth-teller can easily decide whether each information is true or false, the liar has firstly to match each piece of information with his lie and then decide about it. In other words, the greater the number of pieces of information, the greater the liar's cognitive effort to monitor its plausibility (36).

More in depth, the experimental task included nine different types of questions that could be categorized as follows:

Simple Questions (*n* = 30):

- 5 items referred to the experimental condition (EX; e.g., “Are you wearing shoes?”). These are control questions to which all participants are required to respond truthfully.
- 10 items referred to depressive symptoms (DS; e.g., “Do you think more slowly than usual?”).
- 15 items referred to very atypical symptoms (VAS). These questions were taken from the Affective Disorders (AF) scale of the Structured Inventory of Malingered Symptomatology [SIMS; (18); e.g., “Do you rarely laugh?”]. The SIMS is a questionnaire designed to detect malingering through a number of bizarre experiences and highly atypical psychiatric symptoms reported by each participant. The AF scale consists of 15 items about very atypical symptoms of anxiety and depression. An individual is classified as malingering if he/she reports more than five atypical symptoms.
- Complex Questions (*n* = 46):
- 15 items consisted of two *discordant symptoms* (2DS-d): a typical symptom of depression and an atypical symptom of depression (e.g., “Do you face difficulties to concentrate at work, and **are you full of energy?**”).
- 15 items consisted of two *concordant symptoms* (2DS-c); both of them were typical symptoms of depression (e.g., “Do you feel abandoned from the others, and **is your mood sad all day?**”).
- 5 items consisted of two *discordant pieces of information*: a typical depression symptom and an information about the experimental condition (DS&EX-d). One piece of information was correct (in other words, it required a “yes” response), while the other one was not correct (it required a “no” response; e.g., “Do you have difficulties in concentrating, and **are you in Paris?**”).
- 5 items consisted of two *concordant pieces of information*: a typical depression symptom and a piece of information about the experimental condition (DS&EX-c). Both of them are correct (both of them required a “yes” response; e.g., “Are you often sad, and **are you sitting on a chair?**”).
- 3 items consisted of two discordant pieces of information, both of them concerning the experimental condition (2EX-d). One information was correct (in other words, it required a “yes” response), while the other one was not correct (it required a “no” response; e.g., “Are the questions written in red, and **are you wearing shoes?**”).
- 3 items consisted of two concordant pieces of information, both of them concerning the experimental condition (2EX-c). Both of them are correct (both of them required a “yes” response; e.g., “Are you responding with the mouse, and **are you in a room?**”).

The complete list of questions are reported in the Online Supplementary Information. Questions required responding “yes” or “no.” All participants were expected to respond in the same way to the questions concerning the experimental condition (EX) and the very atypical depressive symptoms (VAS). On the contrary, truth-tellers, liars and depressed participants were expected to respond in different ways to the questions, including the depressive symptoms (DS). Indeed, truth-tellers were expected to give 19 “yes” responses and 76 “no” responses in cases in which they denied all depressive symptoms. Depressed participants were expected to give 44 “yes” responses and 57 “no” responses in cases in which they manifested all of the typical depressive symptoms. However, we contemplated that some healthy participants could express few depressive symptoms and, conversely, some depressed patients could deny any of the typical symptoms. For this reason, no feedback was presented in the case of participants who gave an unexpected response (e.g., a healthy participant who responded “yes” to the question “Are you in trouble falling asleep without drugs?”). Finally, liars were expected to give some “yes” responses to depressive symptoms similar to the ones provided by depressed participants. In other words, liars and depressed subjects were expected to declare an equal number of depressive symptoms.

### Experimental procedure

Just before the experimental task, participants assigned to the liar group were instructed to lie about their mood. In particular, they were asked to simulate a depressive status. To increase the compliance, participants were given a little scenario: “*Now, imagine being examined by an insurance policy commission to receive compensation for psychological damage. You have to make them believe that the damage has caused severe depression. So, you have to respond questions simulating a depression, trying to be credible and avoiding being unmasked.”* Conversely, truth-tellers and depressed subjects were asked to answer all the questions truthfully.

**Figure 1:**
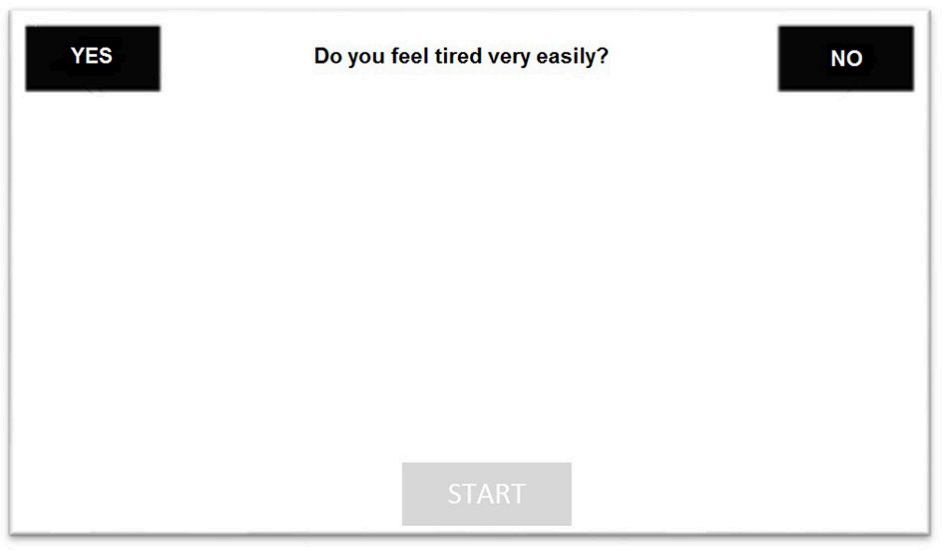
The figure reports an example of the computer screen as appeared to the subjects during the task.

The task was programmed and run using *MouseTracker* software (37). Each participant was presented with 76 randomized questions displayed in the upper part of the computer screen. The squares containing YES and NO response labels were located in the upper left and upper right parts of the screen. Participants were instructed to press the START button (located in the lower part of the screen) to let the questions appear and to then respond to questions by clicking with the mouse on the correct label (YES or NO). Figure 1 shows an example of the computer screen as it appeared to the subjects during the task. The experimental procedure was preceded by 10 training questions, to allow participants to familiarize themselves with the task.

### Data collection

For each answer, motor response was tracked using *MouseTracker* software (37). To permit averaging and comparison across multiple trials, the software performs a time normalization. Specifically, each trajectory is normalized in 101 time frames through linear interpolation. This resulted in each time frame corresponding to specific *x* and *y* coordinates in a binary space. In other words, the software derived the position of the mouse along the axis over the 101 time frames (X_n_,Y_n_). The software also describes the motor response in terms of spatial and temporal features, such as onset, duration, shape, stability and direction of the trajectory. The space–time features recorded by *MouseTracker* are described in detail in Table 1. For each of these features, the average value of the responses in the different types of questions (EX, DS, VAS, 2DS-d, 2DS-c, DS&EX-d, DS&EX-c, 2EX-d, 2EX-c) were computed. In addition, the average velocity (v) and acceleration (a) of the mouse movement between two time frames, respectively, on the *x*-axis (v_*x*_ = X_n_ –X_n−1_ and a_*x*_ = v_x*n*_ – v_x*n*−1_) and *y*-axis (v_*y*_ = Y_n_ – Y_n−1_ and a_*y*_ = v_y*n*_ – v_y*n*−1_) were calculated. The number of symptoms reported by the participants (DS, 2DS-d, 2DS-c, DS&EX-d, DS&EX-c, and VAS) and the number of errors in the control questions related to the experimental condition (EX, 2EX-d, 2EX-c) were also computed. This procedure led to a total of 83 variables that were entered as predictors in machine-learning models (please see the online supplements for a detailed description of the 83 features).

**Table 1:** The table reports the description of the space-time features recorded by *MouseTracker* software.

	**Feature**	**Description**
Temporal features	Initiation time (IT)	Time between the appearance of the question and the beginning of the mouse movement
	Reaction time (RT)	Time from the appearance of the question to the click on the response box
	Maximum deviation time (MD-time)	Time to reach the point of maximum deviation
Spatial features	Maximum deviation (MD)	The largest perpendicular distance between the actual trajectory and the ideal trajectory
	Area under the curve (AUC)	The geometric area between the actual trajectory and the ideal trajectory
	x-flip	Number changes in direction along the *x*-axis
	y-flip	Number changes in of direction along the *y*-axis

## Results

### Visual analysis of mouse trajectories

**Figure 2:**
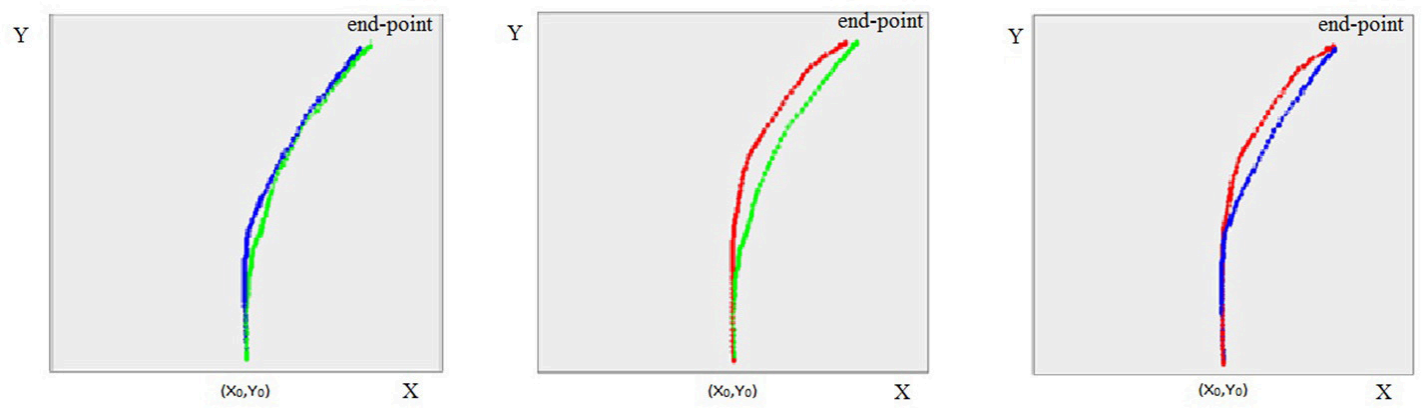
The figure represents the average trajectories between the participants, respectively for liars (in red), truth-tellers (in green) and depressed subjects (in blue), to all questions (EX, DS, VAS, 2DS-d, 2DS-c, DS&EX -d, DS&EX-c, 2 EX-d, 2EX-c).

A preliminary visual analysis was carried out comparing the trajectories of the three experimental groups. Figure 2 compares the average trajectories of liars, truth-tellers and depressed subjects, considering their responses to all the 76 questions. The visual pattern is similar to the one observed in other studies that spot liars through mouse dynamics (26). The trajectories of liars and truth-tellers seem to differ in both AUC and MD parameters. Indeed, both healthy and depressed truth-tellers outlined a more direct trajectory connecting the starting point with the correct response. By contrast, in the initial phase of the response, the liars spent more time moving on the y-axis, and they then deviated toward the response with a delay compared to truth-tellers.

### Univariate statistical analysis

In Table 2, the descriptive statistics of the three experimental groups were reported for the space–time features collected by the software considering all the 76 items of the task.

**Table 2:** The table reports means (M) and standard deviations (SD) for each feature collected by the software.

**Feature**	**Truth-tellers**		**Depressed**		**Liars**	
	***M***	***SD***	***M***	***SD***	***M***	***SD***
IT	620.35	491.94	408.67	332.99	559.57	399.84
RT	4018.79	1466.98	6641.81	3204.22	4030.60	1203.67
MD-time	2392.37	1001.23	4199.85	1818.62	2297.64	620.98
MD	0.44	0.31	0.51	0.30	0.57	0.24
AUC	1.01	0.90	1.09	0.76	1.25	0.67
x-flip	7.64	2.24	8.75	2.94	9.23	2.72
y-flip	7.74	2.63	8.05	2.88	9.20	2.67
v*_*x*_*	0.00627	0.00060	0.00582	0.00076	0.00573	0.00059
v*_*y*_*	0.01326	0.00014	0.01315	0.00010	0.01326	0.00017
a*_*x*_*	−0.00001	0.00004	0.00001	0.00002	0.00000	0.00002
a*_*y*_*	−0.00004	0.00008	−0.00006	0.00004	−0.00001	0.00009

*IT, initiation time; RT, reaction time; MD-time, maximum deviation time; MD, maximum deviation; AUC, area under the curve; x-flip, y-flip, average velocity and acceleration on x and y axis = v_x_, v_y_, a_x_, a_y_, respectively for liars, truth-tellers and depressed subjects responding to all questions*.

A univariate one-way ANOVA was performed on each of the 83 collected features with the aim of identifying the variables that statistically differed between groups. Table 3 reports the variables that differed the three groups.

**Table 3:** The table reports *F*-value, degrees of freedom (*gdl*), *p*-value and effect-size (Omega-squared, ω^2^) resulting from the comparison of the three experimental groups for the features that reached the statistical significance.

**Feature**	**One-way ANOVA (*gdl*, *F-value*, *p-value*, *effect-size*)**
DS	*F*_(2, 57)_ = 93.59, *p* < 0.01, ω = 0.87
EX	*F*_(2, 57)_ = 3.84, *p* < 0.05, ω = 0.29
2DS-d	*F*_(2, 57)_ = 22.94, *p* < 0.01, ω = 0.64
2DS-c	*F*_(2, 57)_ = 91.42, *p* < 0.01, ω = 0.86
DS&EX-c	*F*_(2, 57)_ = 23.49, *p* < 0.01, ω = 0.65
VAS	*F*_(2, 57)_ = 85.6, *p* < 0.01, ω = 0.85
RT	*F*_(2, 57)_ = 9.87, *p* < 0.01, ω = 0.47
RT DS	*F*_(2, 57)_ = 15.22, *p* < 0.01, ω = 0.56
RT EX	*F*_(2, 57)_ = 4.52, *p* < 0.05, ω = 0.32
RT 2DS-d	*F*_(2, 57)_ = 9.24, *p* < 0.01, ω = 0.46
RT 2DS-c	*F*_(2, 57)_ = 11.3, *p* < 0.01, ω = 0.50
RT DS&EX-d	*F*_(2, 57)_ = 7.06, *p* < 0.01, ω = 0.41
RT DS&EX-c	*F*_(2, 57)_ = 7.50, *p* < 0.01, ω = 0.42
RT 2EX-d	*F*_(2, 57)_ = 4.29, *p* < 0.05, ω = 0.31
RT 2EX-c	*F*_(2, 57)_ = 6.06, *p* < 0.01, ω = 0.38
RT VAS	*F*_(2, 57)_ = 7.35, *p* < 0.01, ω = 0.41
MD-time	*F*_(2, 57)_ = 14.68, *p* < 0.01, ω = 0.55
MD-time DS	*F*_(2, 57)_ = 18.25, *p* < 0.01, ω = 0.60
MD-time EX	*F*_(2, 57)_ = 3.25, *p* < 0.05, ω = 0.26
MD-time 2DS-d	*F*_(2, 57)_ = 11.68, *p* < 0.01, ω = 0.51
MD-time 2DS-c	*F*_(2, 57)_ = 14.47, *p* < 0.01, ω = 0.55
MD-time DS&EX-d	*F*_(2, 57)_ = 9.04, *p* < 0.01, ω = 0.45
MD-time DS&EX-c	*F*_(2, 57)_ = 9.2, *p* < 0.01, ω = 0.46
MD-time 2EX-d	*F*_(2, 57)_ = 4.27, *p* < 0.05, ω = 0.31
MD-time 2EX-c	*F*_(2, 57)_ = 12.61, *p* < 0.01, ω = 0.52
MD-time VAS	*F*_(2, 57)_ = 12.62, *p* < 0.01, ω = 0.52
v*_*x*_*	*F*_(2, 57)_ = 3.85, *p* < 0.05, ω = 0.29
v*_*y*_*	*F*_(2, 57)_ = 4.06, *p* < 0.05, ω = 0.30

Finally, a Tukey test was run as a post hoc test to verify which groups accounted for the significant differences found by ANOVA. The results are reported in Table 4.

**Table 4:** Differences between truth-tellers and liars, liars and depressed, truth-tellers and depressed.

**Feature**	**Difference between groups**	**Tukey test, *t*-value, *p*-value**
**TRUTH-TELLERS vs. LIARS**		
DS	−7.75	*t* = −13.38, *p* < 01
2DS-d	5.25	*t* = 6.73, *p* < 0.01
2DS-c	−11.85	*t* = −13.46, *p* < 0.01
DS&EX-c	−2.40	*t* = −6.17, *p* < 0.01
VAS	−6.80	*t* = −13.02, *p* < 0.01
v*_*x*_*	0.00053	*t* = 2.59, *p* < 0.05
**LIARS vs. DEPRESSED**		
DS	2.45	*t* = 4.23, *p* < 0.01
2DS-d	−2.15	*t* = −2.75, *p* < 0.05
2DS-c	5.00	*t* = 5.68, *p* < 0.01
VAS	2.85	*t* = 5.46, *p* < 0.01
RT	−2611.21	*t* = −3.84, *p* < 0.01
RT DS	−2036.49	*t* = −4.79, *p* < 0.01
RT 2DS-d	−3507.4	*t* = −3.89, *p* < 0.01
RT 2DS-c	−2880.60	*t* = −4.14, *p* < 0.01
RT DS&EX-c	−5171.2	*t* = −3.60, *p* < 0.01
RT 2EX-d	−2708.4	*t* = −2.79, *p* < 0.05
RT 2EX-c	−1403.2	*t* = −2.77, *p* < 0.05
RT VAS	−2012.3	*t* = −3.14, *p* < 0.01
MD-time	−1902.21	*t* = −4.80, *p* < 0.01
MD-time DS	−1182.22	*t* = −5.40, *p* < 0.01
MD-time 2DS-d	−2494.9	*t* = −4.39, *p* < 0.01
MD-time 2DS-c	−2076.4	*t* = −4.81, *p* < 0.01
MD-time DS&EX-d	−1741.8	*t* = −2.97, *p* < 0.05
MD-time DS&EX-c	−4187.4	*t* = −3.92, *p* < 0.01
MD-TIME 2EX-d	−1968.1	*t* = −2.88, *p* < 0.05
MD-TIME 2EX-c	−13.45.5	*t* = −4.54, *p* < 0.01
MD-TIME VAS	−1514.67	*t* = −4.35, *p* < 0.01
v*_*y*_*	0.00013	*t* = 2.56, *p* < 0.05
**TRUTH-TELLERS vs. DEPRESSED**		
DS	−5.30	*t* = −9.15, *p* < 0.01
EX	0.45	*t* = 2.72, *p* < 0.05
2DS-d	3.10	*t* = 3.97, *p* < 0.01
2DS-c	−6.85	*t* = −7.78, *p* < 0.01
DS&EX-c	−2.20	*t* = −5.66, *p* < 0.01
VAS	−3.95	*t* = −7.56, *p* < 0.01
RT	−2623.02	*t* = −3.85, *p* < 0.01
RT DS	−2019.08	*t* = −4.75, *p* < 0.01
RT EX	−1027.1	*t* = −3.01, *p* < 0.05
RT 2DS-d	−3183.4	*t* = −3.53, *p* < 0.01
RT 2DS-c	−2836.75	*t* = −4.08, *p* < 0.01
RT DS&EX-d	−3480	*t* = −3.70, *p* < 0.01
RT DS&EX-c	−4350.8	*t* = −3.30, *p* < 0.05
RT 2EX-c	−1621.3	*t* = −3.20, *p* < 0.01
RT VAS	−2225.7	*t* = −3.47, *p* < 0.01
MD-time	−1807.48	*t* = −4.56, *p* < 0.01
MD-time DS	−1100.85	*t* = −5.03, *p* < 0.01
MD-time EX	−560	*t* = −2.50, *p* < 0.05
MD-time 2DS–d	−2240.2	*t* = −3.94, *p* < 0.01
MD-time 2DS-c	−1937.7	*t* = −4.491, *p* < 0.01
MD-time DS&EX-d	−2407.9	*t* = −4.11, *p* < 0.01
MD-time DS&EX-c	−3742.3	*t* = −3.50, *p* < 0.01
MD-time 2EX-c	−1219.7	*t* = −4.12, *p* < 0.01
MD-time VAS	−1510.78	*t* = −4.34, *p* < 0.01

*The table reports t-value, p-value and effect-size resulting from Tukey test and the value of the difference between the compared groups. Only the results that reached the statistical significance are reported*.

### Multivariate analysis: features selection

In order to select the variables to be entered in machine-learning models, a features selection was performed using WEKA 3.9 (38). Features selection is a widely used procedure in machine learning that allows the removal of redundant and irrelevant features and an increase of model generalization by reducing overfitting (39). In the current paper, a correlation-based feature selector (CFS) was used to reduce the number of features (40). This algorithm selects the independent variables with the maximum correlation with the dependent variable (truth-teller vs liar vs depressed) and the minimum correlation across independent variables (the 83 features), using greedy stepwise as search method. The features selected by the CFS are the following: the number of very atypical symptoms (VAS) reported by each participant, the number of depressive symptoms reported by each participant in simple questions (DS), the number of symptoms reported by the participants when they responds to 2DS-c and 2DS-d questions (i.e., complex questions, concordant or discordant, about depressive symptoms), the time needed to reach the point of maximum deviation in 2DS-d questions (MD-time 2DS-d) and the time needed to reach the point of maximum deviation in questions about very atypical symptoms (MD-time VAS). The selected features are reported in Table 5.

**Table 5:** The table reports the 6 features resulted from the features selection.

**Feature**	**Ranked attributes**
DS	0.55
2DS-c	0.52
2DS-d	0.41
VAS	0.52
MD-time 2DS-d	0.36
MD-time VAS	0.37

*The second column reports the value of the correlation between the feature and the dependent variable (truth-teller vs. liar vs. depressed)*.

### Multivariate analysis: machine-learning models

The six features mentioned above were entered in different machine-learning (ML) classifiers. Particularly, we selected four different classifiers that differ for the classification strategy (41–44): Naive Bayes, Sequential Minimal Optimization (SMO), Logistic Model Tree (LMT) and Random Forest (RF). For each classifier, a three-class classification (as the model is required to classify depressed patients, liars and truth-tellers) was run using a 10-fold cross-validation procedure, as implemented in WEKA 3.9 (38). In 10-fold cross-validation, the sample of 60 participants is randomly partitioned into 10 equal size subsamples (n = 6). Of the 10 subsamples, a single subsample is retained as the validation set for testing the model, and the remaining 9 subsamples are used as training sets. The cross-validation process is recursively repeated 10 times, each time with one of the 10 subsamples used as a validation set. The 10 results from the folds are finally averaged to produce a single classification accuracy estimation. The classification accuracies obtained by the four classifiers in 10-fold cross-validation are reported in Table 6. All the classifiers achieved an accuracy ranging from 80 to 90%. In a subsequent step, the four algorithms (ML models) were tested on the group 2 (test = 27 participants) to verify the generalization of the results on an independent sample of participants This allowed us to demonstrate that all the models have a good generalization, as the classification accuracies remain stable at over 90%. The ML results on both the training (*n* = 60) and test (*n* = 27) sets are represented in Table 6.

**Table 6:** Accuracies obtained by four different ML classifiers in 10-fold cross-validation and in test set.

**Classifier**	**Accuracy in 10-fold-cross validation (*n* = 60) (%)**	**Accuracy in test set (*n* = 27) (%)**
Naïve Bayes	90	96.3
SMO	83.3	92.6
LMT	81.6	96.3
Random Forest	80	92.6

*Classifiers are Naïve Bayes, Sequential Minimal Optimization (SMO), Logistic Model Tree (LMT) and Random Forest*.

However, in real world settings, the examiner is required to successfully distinguish malingering from real depression. Thus, the classifications were repeated, entering only liars and depressed participants as classes. In other words, we made a two-class classification and ignored truth-teller participants. The features were selected using the same method described above and then entered in the four classifiers (ML models). The selected features were the following: DS, 2DS-d, 2DS-c, VAS, IT 2EX-d, IT 2EX-c, RT 2EX-c, MD-time, MD-time DS, MD-time 2DS-d, MD-time DS&EX-c, MD-time VAS, a_*y*_, and y-flip DS. Classification accuracies, which were revealed to be stable around 90%, are reported in Table 7.

**Table 7:** Classification accuracies of liars and depressed participants by four different ML classifiers in 10-fold cross-validation and in test set.

**Classifier**	**Accuracy in 10-fold-cross validation (*n* = 60) (%)**	**Accuracy in test set (*n* = 28) (%)**
Naïve Bayes	80	94.4
SMO	82.5	88.9
LMT	80	88.9
Random Forest	87.5	94.4

*Classifiers are Naïve Bayes, Sequential Minimal Optimization (SMO), Logistic Model Tree (LMT) and Random Forest*.

**Figure 3:**
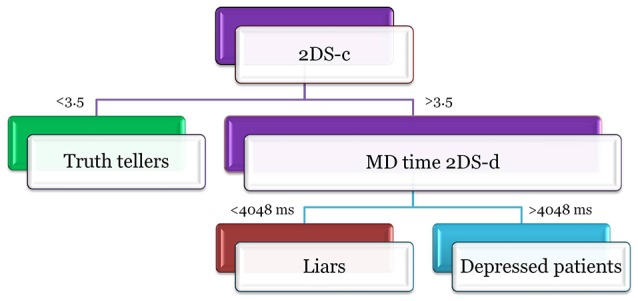
The figure reports the structure of the decision tree model. Participants declaring <3.5 symtomps on 2DS-c questions are classified as truth-tellers. Participants declaring more than 3.5 symtomps on 2DS-c questions are depressed patients if and only if they take more than 4048 ms to compute the response to the 2DS-d questions, otherwise they are classified as liars.

As ML models are difficult to interpret; a decision tree model has been run (45). The decision tree model gives a more simple idea about the hypothetical decision rules, on which the classifications results are based. This is one of the simplest— if not *the* simplest—classifier in terms of transparency of the operations computed by the algorithm, and it permits easy highlighting of the classification logic [even if it is not the most efficient method; (46)]. The structure of the tree is reported in Figure 3. This model is basically built on two rules. The first rule takes into account the number of symptoms declared by the subject in the 2DS-c questions. If the participant reports fewer than 3.5 symptoms, he/she is classified as a truth-teller; else, the second rule is considered. According to the second rule, if the subject takes, on average, more than 4,048 ms to compute the response to the 2DS-d questions, he/she is either a depressed patient or a liar. This simple algorithm reaches an accuracy of 75% in the training group (correctly identifying 51 subjects out of 60), generalizing with an accuracy of 85.2% in the test group.

#### Multivariate analysis: alternative models

One of the most discussed topics in lie detection concerns the resistance to countermeasures (47). If the participant knows how lie detectors work, he/she may enact a series of strategies to reduce its efficacy. For example, an alteration of RTs is enough to beat aIAT or CIT (48, 49), as this is the only parameter on which they are based. In order to prevent countermeasures, the kinematic analysis of mouse movements offers a significant advantage: it is not based simply on RTs but on numerous and articulated parameters that, together, contribute to determine the truthfulness of the subject's response (26). In other words, it would be very difficult for the participants to alter all of the parameters at the same time and keep them under control. Moreover, the large number of features allows the building of alternative classification models. In this way, the examinee cannot know in advance which features are entered in the prediction model and, accordingly, which are the features to keep under control during the test. To fix this point, we developed two alternative machine-learning models, entering in the classifiers a subsets of predictors different from those above used. The six features selected above are the best to optimize the classifier's performance. However, other sub-optimal sets of features can work well in the classification. A first set of alternative predictors contained the five features most correlated to the dependent variable: DS (*r* = 0.55), 2DS-c (*r* = 0.53), VAS (*r* = 0.52), DS&EX-c (*r* = 0.45), MD-time DS (*r* = 0.42). A second subset of predictors included only features related to complex questions about depressive symptoms (2DS-c and 2DS-d), which are the stimuli aimed to increase liars' cognitive load: 2DS-c, 2DS-d, IT 2DS-c, IT 2DS-d, MD-time 2DS-c, MD-time 2DS-d, RT 2DS-c, RT 2DS-d, MD 2DS-c, MD 2DS-d, AUC 2DS-c, AUC 2DS-d, x-flip 2DS-c, x-flip 2DS-d, y-flip 2DS-c, and y-flip 2DS-d. The results obtained from the alternative models are reported in Table 8. It can be noticed that the accuracies remain stable at around 90%, supporting the reliability of this method for the identification of liars.

**Table 8:** Classification of participants using two set of alternative predictors.

**Classifier**	**Accuracy in 10-fold-cross validation (*n* = 60) (%)**	**Accuracy in test set (*n* = 27) (%)**
**SUBSET OF PREDICTORS 1**		
Naïve Bayes	85	88.9
SMO	83.3	88.9
LMT	80	92.6
Random Forest	80	88.9
**SUBSET OF PREDICTORS 2**		
Naïve Bayes	81.6	96.3
SMO	78.3	92.6
LMT	76.7	85.2
Random Forest	75	92.6

*Alternative models were computed using four different ML classifiers (Naïve Bayes, SMO, LMT, Random Forest). Accuracies in 10-fold cross-validation and in test set are reported*.

## Discussion and conclusion

The present study investigated the accuracy of a new deception detection technique in the correct identification of participants who malingered depressive symptoms. To this aim, a tool based on mouse tracking was used while participants were required to answer simple or complex questions concerning both symptoms of depression and the experimental situation.

The main result is striking: the individuals who malingered depressive symptoms were correctly identified by the algorithm with an accuracy of up to 96%. In addition, the current study also underlined that: (i) the mouse trajectory of the liars visually clearly differed from the ones of the truth-tellers (regardless of whether the latter were really depressed); (ii) the group of individuals that malingered depression reported a higher number of depressive and non-depressive symptoms; (iii) the ML classifiers recognized the complex questions within the key features for a correct classification and (iv) liars are also faster than the really depressed subjects—but slower than the healthy truth-tellers—to perform the mouse-based task, as the algorithm identified, as a critical variable for the discrimination, the time to reach the point of maximum deviation during the mouse response.

Importantly, similar results were obtained testing four different ML models (Naïve Bayes, SMO, LTM, Random Forest). This denotes that the results are not highly dependent on the selected algorithm. Furthermore, the main results are obtained using highly selected features, raising the suspicion that they cannot be generalized using different features. This is of outstanding importance, as the number of depressive symptoms (DS) and the number of very atypical symptoms (VAS) were included in the feature selection within the main analysis. Because both DS and VAS could be obtained using simpler tests, as, for instance, the M-test explained in the introduction, one may wonder about the advantage of using the current mouse tracking techniques and whether the current results remain stable even if DS and VAS were removed from the features used for the classification. Critically, these concerns were dampened by the results obtained using alternative ML models, which includes only one (DS) or none of these features within the features selected for the classification. In addition, DS has not been used alone but within the complex sentences. As these alternative models achieved very high classification accuracies as well, this rules out the hypotheses that the current results were driven by the selected features and also sustains the hypothesis that the proposed tools are not easily fooled by coaching. Indeed, the high number of parameters that could be considered to build up the best classifiers and the great variability in the features that could be selected by each classifier makes the new tool ideally suited to be almost impossible to be deceived. Thus, the results reported in the current paper are robust to the ML method and feature selection changes.

It is also worth noting that the tool is based on both mouse tracking movements and the technique of unexpected questions. Contrarily to previously used tests (for instance, the *M*-test), the current algorithm is able to detect the number of symptoms reported by each individual only relying on the use of the complex sentences (2DS-c, 2DS-d), as revealed by the alternative model, and thus excludes potential features that are more easy to be faked, as, for instance, the number of symptoms (DS) and the number of atypical symptoms (VAS).

Concerning the number of symptoms, it should be noted that malingering participants tend to report most of the symptoms which are presented during the task, both typical symptoms of depression (DS) and atypical symptoms (VAS) characterizing other mental disorders. In other words, liars reported being affected by a higher number of psychiatric symptoms than those people who were genuinely depressed. This result is line with literature reporting that the qualitative and quantitative analysis of symptom characteristics is a crucial method to identify simulators (16). It is well known that malingering is often characterized by a positive response to suggested symptoms and a tendency to endorse many symptoms indiscriminately (50). Indeed, malingerers believe that endorsing a symptom will increase the appearance of psychopathology and that more symptoms will be construed as a more severe disorder. On the other hand, genuine patients report only the symptoms that they are really experiencing, resulting in a lower number and more-common symptoms. For this reason, common strategies to detect a malingered response pattern consist in verifying the endorsement of rare and improbable symptoms [e.g., this is how the SIMS works; (18)] or the over-endorsement of symptoms.

The second important piece of evidence concerns the mouse dynamics features. As emerged from the univariate analyses and the algorithm's features selection, the most significant differences between the three experimental groups are in the time to compute the response (RT) and the time to reach the point of maximum deviation (MD-time). In more detail, depressed subjects take more time to respond than the subjects of the other two experimental conditions (liars and truth-tellers) for both simple and complex questions. This result reinforces the evidence available in literature that depression is characterized by psychomotor and ideomotor retardation [diminished ability to think or concentrate, or indecisiveness; (1)]. In other words, depressed people are differentiated from liars not on the basis of the cognitive load, which is higher in liars than in depressed people (who are responding truthfully), but on the basis of the psychomotor retardation, which is a key feature of truly depressed individuals. On the contrary, according to lie-detection literature, liars are slower than healthy truth-tellers, as the greater cognitive load due to the act of lie results in more time needed to compute the response (51).

It is important here to underline that, despite the fact that in literature, it is already known that individuals that feign depression usually report a higher number of symptoms compared to really depressed patients (50) and despite it is already known that lying takes time (28) and thus that liars are, in general, consistently slower than truth-tellers, this study enriches the literature by providing an automatic algorithm that allows combination of the two pieces of information. Critically, this enabled the identification of three different profiles: the non-depressed truth-tellers are characterized by a low number of reported symptoms and by quick answers; the depressed truth-tellers are characterized by a good number of reported symptoms and are very slow in answering and the liars are characterized by a very high number of reported symptoms, and their reaction times are slower than those of the non-depressed truth-tellers but quicker than those of depressed patients.

Finally, two drawbacks are worth highlighting. First, in this paper, the clinician and the machine-learning algorithm performance in detecting malingering has not been compared. Indeed, individuals were selected if they already had a diagnosis of depression. In addition, healthy participants assigned to the truth-tellers or liars groups never underwent a psychiatric examination but were screened using a self-report questionnaire. Thus, we cannot draw definitive conclusions on the superiority of machine learning compared to clinical assessment in detecting the malingering of depression. To date, we can only hypothesize the superiority of machine learning based on previous literature (14, 15). Secondly, eventual cognitive difficulties in patients with depression have not been taken into account. Thus, particular attention should be given to the application of this tool to patients with cognitive disabilities. Those patients could show difficulties both in processing the meaning of the complex questions and in giving the response (they are more likely to drag the mouse while attempting to make a decision, causing distorted trajectories and longer reaction times), obtaining a worse performance than liars. Therefore, the examiner should take into account that the cognitive functioning of the examinee could influence the task performance and thus alter the classifier result. Thus, further studies are needed before this algorithm could be applied to a real-world forensic setting. In particular, this study highlights an urgent need to compare the performance of clinicians and machine learning in detecting malingering, taking into consideration the cognitive difficulties the real patients might be suffering.

In conclusion, we provided evidence that the current algorithm, through an accurate feature selection procedure, can accurately identify up to 96% of the liars. This methodology, compared with the ones currently available in the literature and described in the introduction, has the following advantages: first, this tool is not possible to be cheated on, as there are too many parameters that are taken into consideration; secondly, specialized clinicians are not required to administer it and interpret the results, thus enhancing the possibility of wide use, such as by insurers; thirdly, a single test could be sufficient to understand whether or not an individual is malingering a multifaceted disorder like depression. On the contrary, previous instruments for detecting deception, such as aIAT and CIT, allowed the investigation of a single symptom instead of the disorder itself. Despite the fact that this mouse tracker-based tool has been developed and tested to identify individuals who feigned depression, the same technique could be potentially adapted to allow a wider use and generalization to other psychiatric disorders such as anxiety disorders and PTSD or physical disturbances such as whiplash. Before the translational application to real world forensic setting, further studies are needed to compare the performance of the machine-learning algorithm with the performance of clinicians in detecting malingering.

## Ethics approval and consent to participate

The ethics committee for psychological research of the University of Padova approved the experimental procedure (Unique Number: 276B8771D4B0F6FDC748E0ABE46D460C). All subjects gave written informed consent in accordance with the Declaration of Helsinki.

## Availability of data and materials

The dataset used and analyzed during the current study is available from the corresponding author upon reasonable request.

## Author contributions

MM, GS, and CS: Conceived the experiment; MM and AT: Designed the experimental task; MM and AT: Healthy subjects data acquisition; AT, SF, GT, MV, PD, GP, and TM: Depressed patients data acquisition; MM and GS: Data analysis; MM, GS, and CS: Data interpretation; MM, GS, and CS: Drafting of the manuscript. All the authors revised the manuscript critically and gave the final approval of the version to be published.

### Conflict of interest statement

The authors declare that the research was conducted in the absence of any commercial or financial relationships that could be construed as a potential conflict of interest. The reviewer AH and handling Editor declared their shared affiliation.

## References

[1] American Psychiatric Association DSM V. Diagnostic and Statistical Manual of Mental Disorders. Arlington: American Psychiatric Publishing (2013).

[2] Cuijpers P Smit F. Subclinical depression: a clinically relevant condition? Tijdschr Psychiatr. (2008) 50:519–528. 18688776

[3] Druss BG Rosenheck RA Sledge WH. Health and disability costs of depressive illness in a major U.S. corporation. Am J Psychiatry (2000) 157:1274–8. 10.1176/appi.ajp.157.8.1274 10910790

[4] Ferrari RM,. Breve Manuale di Invalidità Civile. (2016). Available Online at: http://www.raggiungere.it/attachments/article/387/Breve%20Manuale%20di%20Invalidit%C3%A0%20Civile.pdf

[5] Andreani A Tabella danno biologico di lieve entità. (2017). Available Online at: https://www.avvocatoandreani.it/servizi/calcolo_danno_biologico.php

[6] Hayes J Grieve R. Faked depression: comparing malingering via the internet, pen-and-paper, and telephone administration modes. Telemed E Health (2013) 19:714–6. 10.1089/tmj.2012.0278 23870047

[7] Sullivan K King J. Detecting faked psychopathology: a comparison of two tests to detect malingered psychopathology using a simulation design. Psychiatry Res. (2010) 176:75–81. 10.1016/j.psychres.2008.07.013 20116861

[8] Rogers R (2008). Clinical Assessment of Malingering and Deception. ed Rogers R. Guilford Press.

[9] Greve KW Ord JS Bianchini KJ Curtis KL. Prevalence of malingering in patients with chronic pain referred for psychologic evaluation in a medico-legal context. Arch Phys Med Rehabil. (2009) 90:1117–26. 10.1016/j.apmr.2009.01.018 19577024

[10] Mittenberg W Patton C Canyock EM Condit DC. Base rates of malingering and symptom exaggeration. J Clin Exp Neuropsychol. (2002) 24:1094–1102. 10.1076/jcen.24.8.1094.8379 12650234

[11] Young G Psychological injury and law malingering in forensic disability-related assessments: prevalence 15 ± 15%. Psychol Inj Law (2015) 8:188–199. 10.1007/s12207-015-9232-4

[12] Adetunji BA Basil B Mathews M Williams A Osinowo T Oladinni O Detection and management of malingering in a clinical setting. Prim psychiatry (2006) 13:61–69.

[13] Ferrara SD Ananian V Baccino E Boscolo–Berto R Domenici R Hernàndez-Cueto C et al. A novel methodology for the objective ascertainment of psychic and existential damage. Int J Legal Med. (2016) 130:1387–99. 10.1007/s00414-016-1366-8 27147416

[14] Rosenhan D. On being sane in insane places. Science (1973) 179:250–8. 10.1126/science.179.4070.250 4683124

[15] Rosen J Mulsant BH Bruce ML Mittal V Fox D. Actors' portrayals of depression to test interrater reliability in clinical trials. Am J Psychiatry (2004) 161:1909–11. 10.1176/ajp.161.10.1909 15465990

[16] Sartori G Orrù G Zangrossi A Detection of Malingering in personal injury and damage ascertainment. In: Ferrara SD Boscolo-Berto R Viel G editors. Personal Injury and Damage Ascertainment under Civil Law. Springer (2016). pp. 547–58.

[17] Beaber RJ Marston A Michelli J Mills MJ. A brief test for measuring malingering in schizophrenic individuals. Am J Psychiatry (1985) 142:1478–81. 10.1176/ajp.142.12.1478 4073316

[18] Smith GP Burger GK. Detection of malingering: validation of the Structured Inventory of Malingered Symptomatology (SIMS). J Am Acad Psychiatry Law (1997) 25:183–9. 9213290

[19] Storm J Graham JR. Detection of coached general malingering on the MMPI-−2. Psychol. Assess. (2000) 12:158–165. 10.1037/1040-3590.12.2.158 10887761

[20] Agosta S Sartori G. The autobiographical IAT: a review. Front. Psychol. (2013) 4:519. 10.3389/fpsyg.2013.00519 23964261 PMC3741633

[21] Allen JJB Clinical applications of the Concealed Information Test. In: Verschuere B Ben-Shakhar G Meijer E editors. Memory Detection. Theory and Application of the Concealed Information Test. Cambridge: Cambridge University Press (2011). pp. 231–252.

[22] Sartori G Agosta S Gnoato F High accuracy detection of malingered whiplash syndrome. In: International Whiplash Trauma Congress. (Miami, FL) (2007).

[23] Duran ND Dale R McNamara DS. The action dynamics of overcoming the truth. Psychon Bull Rev. (2010) 17:486–491. 10.3758/PBR.17.4.486 20702866

[24] Monaro M Fugazza FI Gamberini L Sartori G How human-mouse interaction can accurately detect faked responses about identity. In: Gamberini L Spagnolli A Jacucci G Blankertz B Freeman J editors. Symbiotic Interaction. Symbiotic 2016. Lecture Notes in Computer Science, Vol 9961. Cham: Springer (2017). pp. 115–124.

[25] Monaro M Gamberini L Sartori G Identity verification using a kinematic memory detection technique. In: Hale K Stanney K editors, Advances in Neuroergonomics and Cognitive Engineering. Advances in Intelligent Systems and Computing, Vol. 488 Cham: Springer (2017). pp. 123–132.

[26] Monaro M Gamberini L Sartori G. The detection of faked identity using unexpected questions and mouse dynamics. PLoS ONE (2017) 12:e0177851. 10.1371/journal.pone.0177851 28542248 PMC5436828

[27] Freeman JB Dale R Farmer TA. Hand in motion reveals mind in motion. Front Psychol. (2011) 2:59. 10.3389/fpsyg.2011.00059 21687437 PMC3110497

[28] Suchotzki K Verschuere B Van Bockstaele B Ben-Shakhar G Crombez G. Lying takes time: a meta-analysis on reaction time measures of deception. Psychol. Bull. (2017) 143:428–453. 10.1037/bul0000087 28182460

[29] Beck AT Ward CH Mendelson M Mock J Erbaugh J. An inventory for measuring depression. Arch Gen Psychiatry (1961) 4:561–571. 10.1001/archpsyc.1961.01710120031004 13688369

[30] Dwork C Feldman V Hardt M Pitassi T Reingold O Roth A. The reusable holdout: preserving validity in adaptive data analysis. Science (2015) 349:3–6. 10.1126/science.aaa9375 26250683

[31] Monaro M Gamberini L Zecchinato F Sartori G. False identity detection using complex sentences. Front Psychol. (2018) 9:283. 10.3389/fpsyg.2018.00283 29559945 PMC5845552

[32] Bertolotti G Michielin P Vidotto G Zotti AM Sanavio E Depression questionnaire (DQ). In: Nezu AM Ronan GF Meadows EA McKlure KS editors. Practitioner's Guide to Empirical Based Measures of Depression. Norwell, MA: Kluwer Academic; Plenum Publishers (2000). pp. 45–47.

[33] Fagiolini A Dell'osso L Pini S Armani A Bouanani S Rucci P et al Validity and reliability of a new instrument for assessing mood symptomatology: the Structured Clinical Interview for Mood Spectrum (SCI-MOODS). Int J Methods Psychiatr Res. (1999) 8:71–82.

[34] Walczyk JJ Igou FP Dixon AP Tcholakian T. Advancing lie detection by inducing cognitive load on liars: a review of relevant theories and techniques guided by lessons from polygraph-Based approaches. Front. Psychol. (2013) 4:14. 10.3389/fpsyg.2013.00014 23378840 PMC3561742

[35] Vrij A Leal S Granhag PA Mann S Fisher RP Hillman J et al. Outsmarting the liars: the benefit of asking unanticipated questions. Law Hum. Behav. (2009) 33:159–166. 10.1007/s10979-008-9143-y 18523881

[36] Williams EJ Bott LA Patrick J Lewis MB. Telling lies: the irrepressible truth? PLoS ONE (2013) 8:e60713. 10.1371/journal.pone.0060713 23573277 PMC3616109

[37] Freeman JB Ambady N. MouseTracker: software for studying real-time mouse-tracking method. Behav Res Methods (2010) 42:226–241. 10.3758/BRM.42.1.226 20160302

[38] Hall MA Frank E Holmes G Pfahringer B Reutemann P Witten IH The WEKA data mining software: an update. ACM SIGKDD Explor. Newslett. (2009) 11:10–18. 10.1145/1656274.1656278

[39] Bermingham ML Pong-Wong R Spiliopoulou A Hayward C Rudan I Campbell H et al. Application of high-dimensional feature selection: evaluation for genomic prediction in man. Sci Rep. (2015) 5:1–12. 10.1038/srep10312 25988841 PMC4437376

[40] Hall MA (1999). Correlation-based Feature Selection for Machine Learning. The University of Waikato, Hamilton.

[41] Breiman L Random forest. Mach. Learn. (2001) 45:5–32. 10.1023/A:1010933404324

[42] John GH Langley P Estimating continuous distributions in Bayesian classifiers. In: Proceeding of the 11th Conference on Uncertainty in Artificial Intelligence. San Mateo, CA (1995). pp. 338–345.

[43] Keerthi SS Shevade SK Bhattacharyya C Murthy KRK Improvements to platt's SMO algorithm for SVM classifier design. Neural Comput. (2001) 13:637–649. 10.1162/089976601300014493

[44] Landwehr N Hall M Frank E Logistic model trees. Mach Learn. (2005) 95:161–205. 10.1007/s10994-005-0466-3

[45] Quinlan JS (1993). C4.5: Programs for Machine Learning. San Mateo, CA: Morgan Kaufmann Publishers.

[46] Mitchell T Decision tree learning. In: Machine Learning. Mitchell T editor. New York, NY: McGraw Hill (1997). pp. 52–78.

[47] Bowman H Filetti M Alsufyani A Janssen D Su L. Countering countermeasures: detecting identity lies by detecting conscious breakthrough. PLoS ONE (2014) 9:e90595. 10.1371/journal.pone.0090595 24608749 PMC3946631

[48] Agosta S Ghirardi V Zogmaister C Castiello U Sartori G Detecting fakers of the autobiographical IAT. Appl Cogn Psychol. (2010) 25:299–306. 10.1002/acp.1691

[49] Peth J Suchotzki K Matthias G. Influence of countermeasures on the validity of the concealed information test. Psychophysiology (2016) 53:1429–40. 10.1111/psyp.12690 27338719

[50] Conroy MA Kwartner PP Malingering. Appl Psychol Crim Justice (2006) 2:29–51.

[51] Monaro M Galante C Spolaor R Li QQ Gamberini L Conti M et al. Covert lie detection using keyboard dynamics. Sci Rep. (2018) 8:1976. 10.1038/s41598-018-20462-6 29386583 PMC5792443

