# Supplementary material for: The Detection of Malingering: A New Tool to Identify Made-Up Depression
Source: Front Psychiatry. 2018 Jun 8;9:249. doi: 10.3389/fpsyt.2018.00249 (PMC6002526; doi:10.3389/fpsyt.2018.00249)
Supplement: Supplementary file 1 [file Table_1.PDF]

Supplementary Material

The detection of malingering: a new tool to identify made up depression

Merylin Monaro, Andrea Toncini, Stefano Ferracuti, Gianmarco Tessari, Maria Grazia Vaccaro, Pasquale De Fazio, Giorgio Pigato, Tiziano Meneghel, Cristina Scarpazza*, Giuseppe Sartori

*** Correspondence:** Corresponding Author: cristina.scarpazza@gmail.com

The complete list of questions presented to the subjects during the task.

The first column reports the text of the questions, whereas the second and the third columns report respectively the response expected by a subject who declare to be affected by depression (liars and depressed patients) and the response expected by a healthy subject (truth tellers).

**Questions**	**Depression Response (liars and depressed patients)**	**Healthy Response (truth tellers)**
**Training questions**		
Are you carrying out a questionnaire?	YES	YES
Are you sitting on a chair?	YES	YES
Are you climbing a mountain?	NO	NO
Have you ever been to the Moon?	NO	NO
Have you ever wore sneakers?	YES	YES
Are you in a town and **are you dressed?**	YES	YES
Are you in France and **are you reading some questions?**	NO	NO
Are you in front of a computer?	YES	YES
Are you relaxed and **are you sitting on a chair?**	NO	YES
Do you feel depressed and **have you often tingling in the fingers?**	YES	NO
**DS**		
Are you in trouble falling asleep without drugs?	YES	NO
Do you think slower than usually?	YES	NO
Have you more difficulties to concentrate on work?	YES	NO
Do you often want to cry?	YES	NO
Do you feel tired very easily?	YES	NO
Have you a normal appetite?	NO	YES
Is your interest for sex decreased?	YES	NO
Do you feel a burden to others?	YES	NO

Does your life deserve to be lived?	NO	YES
Do you complain a lot?	YES	NO
**EX**		
Are you sitting on a sofa?	NO	NO
Are you in a room?	YES	YES
Are you wearing shoes?	YES	YES
Are you shirtless?	NO	NO
Are you alone in the room?	NO	NO
**2DS-d**		
Do you mind taking a decision and **are you always focused on what you do?**	NO	NO
Are you depressed all day long and **do you want to do more things during the day?**	NO	NO
Is your interest for things decreased and **do you eat as usually?**	NO	NO
Do you complain a lot and **do you have many hobbies?**	NO	NO
Do you have a normal appetite and **do you have less sexual interest?**	NO	NO
Do you face difficulties to concentrate at work and **are you full of energy?**	NO	NO
Do you feel to cry and **laugh at the same time?**	NO	NO
Are you very critical and **sociable to others?**	NO	NO
Are you always sad and **do you hear voices in your head?**	NO	NO
Do you have nightmares about your past and **do you feel always tired?**	NO	NO
Do you have less appetite and **do you wash your hands continuously?**	NO	NO
Do you see things that others can’t see and **do you feel weak all day?**	NO	NO
Are you devoted to mystical meditations and **have you lost interest in others' company?**	NO	NO
Does it feel that time doesn’t pass and **do you spend too much money?**	NO	NO

Do you always want to stay at the center of attention and **do you complain a lot?**	NO	NO
**2DS-c**		
Do you feel abandoned from the others and **is your mood sad all day?**	YES	NO
Do you think that the future will not reserve you any good and **would you like to get it over?**	YES	NO
Do you feel that the time never pass and **are you always tired?**	YES	NO
Are you always weak and **depressed only in the morning?**	YES	NO
Do you think more slowly than usual and **do you feel sad?**	YES	NO
Do you have always dry-throat and **do you have less appetite than usual?**	YES	NO
Do you have difficulty falling asleep and **do you have little appetite?**	YES	NO
Do you feel a burden to others and **would you like to die?**	YES	NO
Do you often cry and **are you inconsolable?**	YES	NO
Do you often complain and **are you very critical?**	YES	NO
Do you have difficulty getting out of bed and **do you have many hobbies?**	YES	NO
Do you often feel like crying and **do you have less sexual interest?**	YES	NO
Is your thinking slowed down and **are you always weak?**	YES	NO
Do you have difficulties in concentrating and **don't you want to work?**	YES	NO
Don't you have interest in others and **don't you want to go running?**	YES	NO
**DS&EX-d**		
Are you climbing a mountain and **do you have bad thoughts about the future?**	NO	NO
Do you have difficulties in concentrating and **are you in Paris?**	NO	NO

Do all foods have the same taste and **are you shirtless?**	NO	NO
Is there anyone with you in the room and **do you have many hobbies?**	NO	YES
Are you sitting on a sofa and **do you have strong feeling of guilt?**	NO	NO
**DS&EX-c**		
Are you often sad and **are you sitting on a chair?**	YES	NO
Do you often criticize the others and **are you in front of a computer?**	YES	NO
Don't you care to make new friends and **are you in a room?**	YES	NO
Doesn't the life worth living and **are you wearing shoes?**	YES	NO
Are you performing an experiment and **have you less sexual interest?**	YES	NO
**2EX-d**		
Are the questions written in red and **are you wearing shoes?**	NO	NO
Are you in the countryside and **is the door closed?**	NO	NO
Are you in a laboratory and **are you using a pen?**	NO	NO
**2EX-c**		
Are you responding with the mouse and **are you in a room?**	YES	YES
Are you in Italy and **are you in front of a computer?**	YES	YES
Are you reading questions and **is there someone else in the room?**	YES	YES
**VAS**		
Do you feel better in the morning after a good night's sleep?	YES	YES
Is your mood getting worse during the day?	YES	YES
Can't you express your feeling?	YES	YES
Do you rarely laugh?	YES	YES

Is your mood worse at night?	YES	YES
Are you always depressed?	YES	YES
Do you rarely cry?	NO	NO
Do you feel better thanks to hobbies or interests or friends?	NO	NO
If you are too depressed would you come out to take exercise to reduce tension?	YES	YES
Don't you seem to have the same energy compared to the past?	YES	YES
Do you remain hopeful even when things are bad?	NO	NO
Do you have trouble sleeping?	YES	YES
Sometimes do you feel so depressed that gladly go to bed early to sleep on it?	NO	NO
Do you often wake up at night despite not having trouble falling asleep?	YES	YES
Do you eat more when you feel more depressed?	YES	YES

The complete list of predictors that were entered as predictors in machine learning models.

The table reports the label of the variable (first column) its description (second column).

Variable	Description
DS	Score to simple questions about depressive symptoms
EX	Score to simple questions about experimental condition
VAS	Score to simple questions about atypical depressive symptoms
2DS-d	Score to complex questions containing two discordant symptoms
2DS-c	Score to complex questions containing two concordant symptoms
DS&EX-d	Score to complex questions containing a symptom and a detail about the experimental condition which require discordant responses
DS&EX-c	Score to complex questions containing a symptom and a detail about the experimental condition which require concordant responses
2EX-d	Score to complex questions containing two discordant details about the experimental condition
2EX-c	Score to complex questions containing two concordant details about the experimental condition
IT	Average initiation time for all questions
IT DS	Average initiation time for DS questions

IT EX	Average initiation time for EX questions
IT 2DS-d	Average initiation time for the first move after pressing START for the variable 2DS-d
IT 2DS-c	Average initiation time for 2DS-c questions
IT DS&EX-d	Average initiation time for DS&EX-d questions
IT DS&EX-c	Average initiation time for DS&EX-c questions
IT 2EX-d	Average initiation time for 2EX-d questions
IT 2EX-c	Average initiation time for 2EX-c questions
IT VAS	Average initiation time for VAS questions
RT	Average reaction time to all questions
RT DS	Average reaction time for DS questions
RT EX	Average reaction time for EX questions
RT 2DS-d	Average reaction time for 2DS-d questions
RT 2DS-c	Average reaction time for 2DS-c questions
RT DS&EX-d	Average reaction time for DS&EX-d questions
RT DS&EX-c	Average reaction time for DS&EX-c questions
RT 2EX-d	Average reaction time for 2EX-d questions
RT 2EX-c	Average reaction time for 2EX-c questions
RT VAS	Average reaction time for VAS questions
MD	Average maximum deviation for all questions
MD DS	Average maximum deviation for DS questions
MD EX	Average maximum deviation for EX questions
MD 2DS-d	Average maximum deviation for 2DS-d questions

MD 2DS-c	Average maximum deviation for 2DS-c questions
MD DS&EX-d	Average maximum deviation for DS&EX-d questions
MD DS&EX-c	Average maximum deviation for DS&EX-c questions
MD 2EX-d	Average maximum deviation for 2EX-d questions
MD 2EX-c	Average maximum deviation for 2EX-c questions
MD VAS	Average maximum deviation for VAS questions
AUC	Average area under the curve for all questions
AUC DS	Average area under the curve for DS questions
AUC EX	Average area under the curve for EX questions
AUC 2DS-d	Average area under the curve for 2DS-d questions
AUC 2DS-c	Average area under the curve for 2DS-c questions
AUC DS&EX-d	Average area under the curve for DS&EX-d questions
AUC DS&EX-c	Average area under the curve for DS&EX-c questions
AUC 2EX-d	Average area under the curve for 2EX-d questions
AUC 2EX-c	Average area under the curve for 2EX-c questions
AUC VAS	Average area under the curve for VAS questions

MD-time	Average maximum deviation time for all questions
MD-time DS	Average maximum deviation time for DS questions
MD-time EX	Average maximum deviation time for EX questions
MD-time 2DS-d	Average maximum deviation time for 2DS-d questions
MD-time 2DS-c	Average maximum deviation time for 2DS-c questions
MD-time DS&EX-d	Average maximum deviation time for DS&EX-d questions
MD-time DS&EX-c	Average maximum deviation time for DS&EX-c questions
MD-time 2EX-d	Average maximum deviation time for 2EX-d questions
MD-time 2EX-c	Average maximum deviation time for 2EX-c questions
MD-time VAS	Average maximum deviation time for VAS questions
x-flip	Average number of changes in direction along the x-axis for all the questions
x-flip DS	Average number of changes in direction along the x-axis for DS questions
x-flip EX	Average number of changes in direction along the x-axis for EX questions

x-flip 2DS-d	Average number of changes in direction along the x-axis for 2DS-d questions
x-flip 2DS-c	Average number of changes in direction along the x-axis for 2DS-c questions
x-flip DS&EX-d	Average number of changes in direction along the x-axis for DS&EX-d questions
x-flip DS&EX-c	Average number of changes in direction along the x-axis for DS&EX-c questions
x-flip 2EX-d	Average number of changes in direction along the x-axis for 2EX-d questions
x-flip 2EX-c	Average number of changes in direction along the x-axis for 2EX-c questions
x-flip VAS	Average number of changes in direction along the x-axis for VAS questions
y-flip	Average number of changes in direction along the y-axis for all the questions
y-flip DS	Average number of changes in direction along the y-axis for DS questions
y-flip EX	Average number of changes in direction along the y-axis for EX questions
y-flip 2DS-d	Average number of changes in direction along the y-axis for 2DS-d questions
y-flip 2DS-c	Average number of changes in direction along the y-axis for 2DS-c questions
y-flip DS&EX-d	Average number of changes in direction along the y-axis for DS&EX-d questions

y-flip DS&EX-c	Average number of changes in direction along the y-axis for DS&EX-c questions
y-flip 2EX-d	Average number of changes in direction along the y-axis for 2EX-d questions
y-flip 2EX-c	Average number of changes in direction along the y-axis for 2EX-c questions
y-flip VAS	Average number of changes in direction along the y-axis for VAS questions
Vel x	Average velocity along x axis for all the questions
Vel y	Average velocity along y axis for all the questions
Acc x	Average acceleration along x axis for all the questions
Acc y	Average acceleration along y axis for all the questions
